# Physiological and transcriptional responses to heat stress and functional analyses of *PsHSP*s in tree peony (*Paeonia suffruticosa*)

**DOI:** 10.3389/fpls.2022.926900

**Published:** 2022-08-11

**Authors:** Jin Ma, Jie Wang, Qun Wang, Linxue Shang, Yu Zhao, Guozhe Zhang, Qingqing Ma, Sidan Hong, Cuihua Gu

**Affiliations:** ^1^Zhejiang Provincial Key Laboratory of Germplasm Innovation and Utilization for Garden Plants, Key Laboratory of National Forestry and Grassland Administration on Germplasm Innovation and Utilization for Southern Garden Plants, College of Landscape and Architecture, Zhejiang Agriculture and Forestry University, Hangzhou, China; ^2^Shenzhen Branch, Guangdong Laboratory for Lingnan Modern Agriculture, Genome Analysis Laboratory of the Ministry of Agriculture, Agricultural Genomics Institute at Shenzhen, Chinese Academy of Agricultural Sciences, Shenzhen, China; ^3^Kunpeng Institute of Modern Agriculture at Foshan, Foshan, China

**Keywords:** high temperature, abiotic, comparative transcriptomics, peony cultivar, transgenic line

## Abstract

Tree peony (*Paeonia suffruticosa*) is a traditional Chinese flower that is not resistant to high temperatures, and the frequent sunburn during summer limits its normal growth. The lack of understanding of the molecular mechanisms in tree peony has greatly restricted the improvement of novel heat-tolerant varieties. Therefore, we treated tree peony cultivar “Yuhong” (*P. suffruticosa* “Yuhong”) at normal (25°C) and high temperatures (40°C) and sequenced the transcriptomes, to investigate the molecular responsive mechanisms to heat stress. By comparing the transcriptomes, a total of 7,673 differentially expressed genes (DEGs) were detected comprising 4,220 upregulated and 3,453 downregulated genes. Functional annotation showed that the DEGs were mainly related to the metabolic process, cells and binding, carbon metabolism, and endoplasmic reticulum protein processing. qRT-PCR revealed that three *sHSP* genes (*PsHSP17.8*, *PsHSP21*, and *PsHSP27.4*) were upregulated in the response of tree peony to heat stress. Tissue quantification of the transgenic lines (*Arabidopsis thaliana*) showed that all three genes were most highly expressed in the leaves. The survival rates of transgenic lines (*PsHSP17.8*, *PsHSP21*, and *PsHSP27.4*) restored to normal growth after high-temperature treatment were 43, 36, and 31%, respectively. In addition, the activity of superoxide dismutase, accumulation of free proline, and chlorophyll level was higher than those of the wild-type lines, while the malondialdehyde content and conductivity were lower, and the membrane lipid peroxidation reaction of the wild-type plant was more intense. Our research found several processes and pathways related to heat resistance in tree peony including metabolic process, single-organism process, phenylpropane biosynthesis pathway, and endoplasmic reticulum protein synthesis pathway. *PsHSP17.8*, *PsHSP21*, and *PsHSP27.4* improved heat tolerance by increasing SOD activity and proline content. These findings can provide genetic resources for understanding the heat-resistance response of tree peony and benefit future germplasm innovation.

## Introduction

Tree peony (*Paeonia suffruticosa* Andrews, Paeoniaceae) is a type of perennial deciduous subshrub originating in China as one of the 10 traditional Chinese flowers (Chen, 1999). The colors of cultivated peony range from red, purple, yellow, green, pink, and white, to multi-color, and the corolla can be divided into many types such as single-petal type, lotus type, chrysanthemum type, and pavilion type, according to the shape and layer of petals and degrees of petalody of the pistils and stamens (Zhou et al., 2014). Therefore, tree peony has been widely cultivated for its high ornamental value. Cultivated populations of tree peony are widely distributed across the temperate regions of China (Zhou, 2006). According to the geographical locations of the cultivated centers, more than 1,000 tree peony cultivars have been divided into four groups, namely the Xibei, Xinan, Zhongyuan, and Jiangnan groups (Hong and Pan, 1999; Ji et al., 2012), of which the Jiangnan group tends to have shallower roots and a higher plant height with more branches. Jiangnan group also showed higher resistance to humidity and heat. However, most of the other tree peony cultivars are subjected to mild and cool climates and are weakly resistant to high temperatures (Zhang et al., 2007), which often grow poorly and even go dormant, damaging ornamental effects and application scopes, especially in the dilemma caused by increasing global warming.

Under high temperatures, plants show a variety of survival changes, including morphological, physiological, and biochemical changes (Mittler et al., 2012; Ohama et al., 2017). For example, the lipids and cell membrane fluidity will change under heat stress, and the Ca^2+^ channels will gradually activate several signal transduction pathways involving phosphatases, reactive oxygen species (ROS), kinases, transcription factors (TFs), and hormones to regulate changes in the transcriptome, proteome, and metabolome (McClung and Davis, 2010; Suzuki et al., 2012). The reproduction and survival of plants in a high-temperature environment depend on sensing temperature stimuli and producing and transmitting signals, thereby triggering changes in the corresponding physiological and biochemical processes to increase plant tolerance to heat (Hasanuzzaman et al., 2013). For instance, an abnormally high temperature increases the fluidity and permeability of cell membranes and denatures proteins. Environmental signals are transmitted to trigger transduction pathways, of which the most obvious response is the Ca^2+^ accumulation and flow from the extracellular matrix into the cell (Vigh et al., 2007; Wu and Jinn, 2010; Niu and Xiang, 2018). Additionally, high temperatures disturb the accumulation of activated oxygen species (AOS), such as malondialdehyde (MDA), superoxide radical (O_2_^−^), and hydroxyl (−OH), which results in peroxidative damage to lipids, nucleic acids, proteins, and cell membranes (McClung and Davis, 2010; Mittler et al., 2012; Suzuki et al., 2012). Plants will mobilize a variety of antioxidant enzymes to slow down lipid membrane peroxidation. The accumulation of osmotic regulating substances, such as carbohydrates, proline, and soluble proteins, can strengthen the stability of proteins and membranes (Park et al., 2007; Qu et al., 2013). Many hormones, including ethylene (ET), salicylic acid (SA), abscisic acid (ABA), jasmonic acid (JA), and auxin (AUX), also improve stress resistance in plants (Miller et al., 2008; Mittler et al., 2012; Qu et al., 2013).

Proteins tend to denature, aggregate, and degrade under heat stress, inducing the upregulated expression of many heat shock proteins (HSPs) to protect cells and transduce signals (Huang and Xu, 2008; Xie et al., 2020). HSPs can be generally divided into five families according to molecular weight, i.e., HSP100/ClpB family proteins, HSP90 family proteins, HSP70/Dnak family proteins, HSP60/GroEl molecular chaperones, and small heat shock proteins (sHSPs)/HSP20 (Sun et al., 2002; Waters, 2012; Xie et al., 2020). These HSPs synergistically participate in cellular protein folding and protect vital functional activities from being affected. For example, HSP70 and HSP90 can be activated by stimulatory signals to synthesize HSP chaperones and other stress proteins. HSP70 and sHSP can stabilize proteins and suppress abnormal aggregation. HSP90, HSP70, and HSP60 can act on protein refolding, and HSP100 can dissolve abnormal aggregation (Waters and Vierling, 2020). In particular, sHSPs can stabilize non-native proteins. They function in the normal physiological activities of stabilizing cell membranes and preventing abnormal excessive aggregation and decay of proteins (Xie et al., 2020). They often coordinate with HSP60, HSP70, or HSP100 members involved in protein activation, transport, or refolding (Bondino et al., 2012).

Since few studies have focused on the response of tree peony to heat stress, the mechanisms underlying the response to heat stress remain largely unknown. To explore the molecular mechanism of heat resistance in tree peony, the transcriptomes of *P. suffruticosa* “Yuhong” seedlings treated at normal and high temperatures were sequenced to scan differential *HSP* genes. The functions of the three *PsHSP* genes were analyzed using transgenic *Arabidopsis thaliana*. Our results will provide genetic resources for germplasm innovation of tree peony and the future breeding of high heat-resistant cultivars.

## Materials and methods

### Plant materials and heat stress treatment

*Paeonia suffruticosa* “Yuhong” is one of the high heat-resistant cultivars in the Jiangnan group grown in the intelligent experimental shed of Zhejiang A&F University (119°72′ E, 30°23′ N). Six annual seedlings with the same growth were individually transplanted into single flowerpots with normal water and fertilizer management and then placed into the growth cabinet RXZ-100A (Dongnan Instruction Co., LTD., Ningbo, China) at 25°C, with 12 h/12 h light/dark, 4,000 lx light intensity, and 80% humidity for 2 weeks for pretreatment. Subsequently, according to previous research (Liu and Chu, 2015; Ding et al., 2021) and the limits of local climate conditions, the three samples of the heat stress treatment group (TE) were placed in the growth cabinet at 40°C for 36 h, and the other three samples of the control group (CK) were treated at a temperature of 25°C. Three biological replicates were set for each group. The leaves of each sample were collected from the same position on the stem. The morphological samples after 24 h of stress treatment were collected for RNA sequencing (RNA-seq). Leaves were immediately frozen in liquid nitrogen and then stored at −80°C.

### Total RNA extraction, sequencing, and assembly

The total RNA of “Yuhong” leaves was extracted using a Trizol kit (Tiangen Biotech Co., LTD., Beijing, China), and the quality was checked by an ultraviolet spectrophotometer and 1.5% agarose gel electrophoresis. The pair-end complementary DNA (cDNA) library was constructed and then sequenced using Illumina HiSeq2500 (Biomac Biotechnology Co., Ltd., Beijing, China), with an insert size of 250 bp based on sequencing by synthesis (SBS) technology to generate the raw data. Trimmomatic v0.39 (Bolger et al., 2014) and Perl script were used for quality control of the raw data, including the truncation of adapters and primer sequences and filtration of low-quality reads to obtain high-quality clean reads. Due to the lack of reference genomes, Trinity v2.1.1 (Grabherr et al., 2011) was used for parameter-free assembly to obtain the transcripts. The redundant transcripts (similarity >90% and overlap length > 35 bp) were integrated and clustered using cd-hit v4.8.1 (Fu et al., 2012). Among the different isoforms of the same gene, the longest was selected as a unigene. Finally, the clean data of each sample were mapped back against the assembled UniGene database to assess assembly quality.

### Annotation, enrichment, and bioinformatic analyses

The clean reads were aligned against the UniGene database using Bowtie2 v 2.4.2 (Langmead and Salzberg, 2012), and fragments per kilobase of exon model per million mapped fragments (FPKM) were used to measure gene expression. The protein sequences of unigenes were aligned to eight databases, i.e., Non-Redundant Protein Sequence Database (NR), Swiss-Prot, Eggnog v4.5, Gene Ontology (GO), Cluster of Orthologous Groups of Proteins (COG), Clusters of Orthologous Groups for Eukaryotic Complete Genomes (KOG), Protein family (Pfam), and Kyoto Encyclopedia of Genes and Genomes (KEGG), to obtain annotations. The differentially expressed genes (DEGs) between CK and TE were screened using DESeq2 v1.3.1 (Love et al., 2014) based on log_2_|fold change (FC)| ≥ 1 and false discovery rate (FDR) < 0.01 and then enriched in GO function and the KEGG pathway. DNAMAN v7^1^ was used for the sequence alignment of *PsHSP* genes, and BioXM v2.7.1 (Cirillo et al., 2017) was used to detect the restriction enzyme cutting sites. PHYRE v2.0 (Kelley et al., 2015) was used for the simulation of the protein hierarchical structure and MEGA v7 (Kumar et al., 2016) for phylogenetic reconstruction. ProtParam (Gasteiger et al., 2005) and TMHMM-2.0^2^ were used for amino acid physicochemical property classification, protein structure prediction, and secondary coiled-coil and gene functional analyses.

### Quantitative real-time PCR

For real-time quantitative polymerase chain reaction (qRT-PCR), the reaction mix contained 3.2 μl ddH_2_O, 0.4 μl upstream primer, 0.4 μl downstream primer, 1 μL cDNA template, and 5 μl SYBR Taq enzyme. The settings for qRT-PCR were as follows: 95°C for 30 s, 95°C for 5 s, and 60°C for 30 s, with a total of 38 cycles. The relative expression of the target gene was calculated by the 2^-ΔΔCt^ method (Arya et al., 2005) using the *Ubiquitin* gene as the reference. Samples of leaves, flowers, siliques, and stems of plants under normal conditions were collected and frozen in liquid nitrogen for tissue quantification. The protocol of qRT-PCR was the same as above but with the *Atβ-actin* gene as the reference. Three biological replicates were set during the experiment.

### Sequence obtainment and transgenic lines of *PsHSP*

Total RNA from the leaves was used to synthesize complementary DNA (cDNA) sequences of *PsHSP17.8*, *PsHSP21*, and *PsHSP27.4*, which were amplified by PCR with designed primers. To further investigate the role of these three *sHSPs* in tree peony resistance to heat stress, double digestion (*Xho* I and *Cla* I) was used to construct the expression vectors (AY562547.1) of the three *sHSPs* (Supplementary Figure S1). *Agrobacterium*-mediated transformation (GV3101) was used for transgenic *A. thaliana*. With reference to Hong and Vierling (2000) and Tiwari et al. (2020), the seeds were sown with antibiotics containing 100 mg·mL^−1^ kanamycin +50 mg·mL^−1^ timentin +50 mg·mL^−1^ ampicillin in 1/2 MS solid medium (Supplementary Figures S2, S3). They were placed in the dark at 4°C for 2 days and then transferred to the tissue culture room. The seedlings were grown for 10 days, selected, and transplanted into the nutrient soil matrix with one plant per pot. Selection was repeated on the resistant medium until the T_3_ generation. Then, the seeds of the WT and T_3_ transgenic lines were washed, sterilized, and sown, and three replicates with about 100 seeds in each were set. After 10 days in the growth cabinet under 25°C, 12 h/12 h light/dark, 4,000 lx light intensity, and 80% humidity conditions, all seedlings were exposed to 40°C for 4 h with 4,000 lx light intensity and 75% humidity. After 21 days of recovery at 25°C, overall growth was observed.

### Measurements of physiological indices

The MT and T_3_ transgenic lines were stressed at 40°C for 12 h, and then, leaves from the same position were immediately collected to measure relevant physiological indices. Chlorophyll content was extracted by macerating in 80% acetone and determined with a spectrophotometer (UV-2550, Shimadzu, Tokyo, Japan), and the conductivity of the leaves was measured by a conductivity bridge (DDSJ-308A, Shanghai, China) in accordance with Shen et al. (2017). Superoxide dismutase (SOD) activity was measured using the nitro-blue tetrazolium (NBT) method. Proline content was determined with a ninhydrin reaction, and MDA content was assayed using the thiobarbituric acid (TBA) method, as described in Chang et al. (2020). All the indices of each sample were measured with three biological and technical replicates.

## Results

### Phenotypic changes

**Figure 1:**
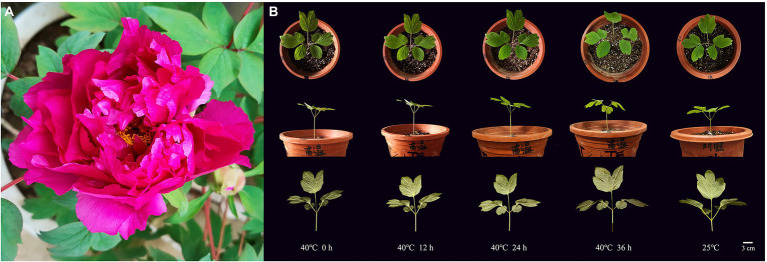
Plant materials of tree peony “Yuhong.” **(A)** Ornamental flowers. **(B)** Phenotypic changes of “Yuhong” under heat stress at 40°C for 0–36 h and CK.

Obvious phenotypic changes in the leaves were observed with an increasing time under heat stress at 40°C (Figure 1). At 0 h, the plant was erect; the leaves were flat without abnormal spots or burn marks on the surface and back, and the color was emerald green (Figure 1A). After 12 h, the plant did not show significant changes, but some shallow brown burn marks appeared sporadically on the back of the leaves. After 24 h of heat stress, the plant was still upright. Small black spots appeared on the leaf surface. The burn marks on the back of the leaves expanded, and the leaves began to droop. After 36 h of stress, the stem remained upright, the leaves drooped from the top and gradually faded green from the margin, and the color of the burn marks deepened from light brown to dark brown. The burn area expanded more than before. The phenotype of CK at 25°C did not change obviously within 36 h (Figure 1B).

### Transcriptome overview and DEG analysis

A total of 45.97 Gb of RNA-seq clean reads were generated after quality control for six samples from the control group (CK_1_, CK_2_, and CK_3_) and the treatment group (TE_1_, TE_2_, and TE_3_). The number of clean bases in each sample was 6.86–9.08 Gb, and the count of clean reads ranged from 22,967,108 to 30,430,627. The GC content was stable at 43.68–44.96%, and the proportion of Q30 bases in each sample was ≥96%. All unigenes were blasted against the eight databases to obtain annotations. A total of 24,309 unigenes were annotated, of which 9,731 (40.03%) unigenes were 300–1,000 bp, and 14,578 (59.97%) were over 1,000 bp. Among the annotated unigenes, the most annotations were obtained in NR (24,009; 98.76%), Eggnog (21,798; 89.67%), and Swiss-Prot (15,426; 63.46%), while the least were from COG (6,915; 28.44%). There were 15,108 (62.15%), 14,568 (59.93%), 13,669 (56.23%), and 8,416 (34.62%) unigenes annotated in Pfam, GO, KOG, and KEGG, respectively (Supplementary Figure S4).

**Figure 2:**
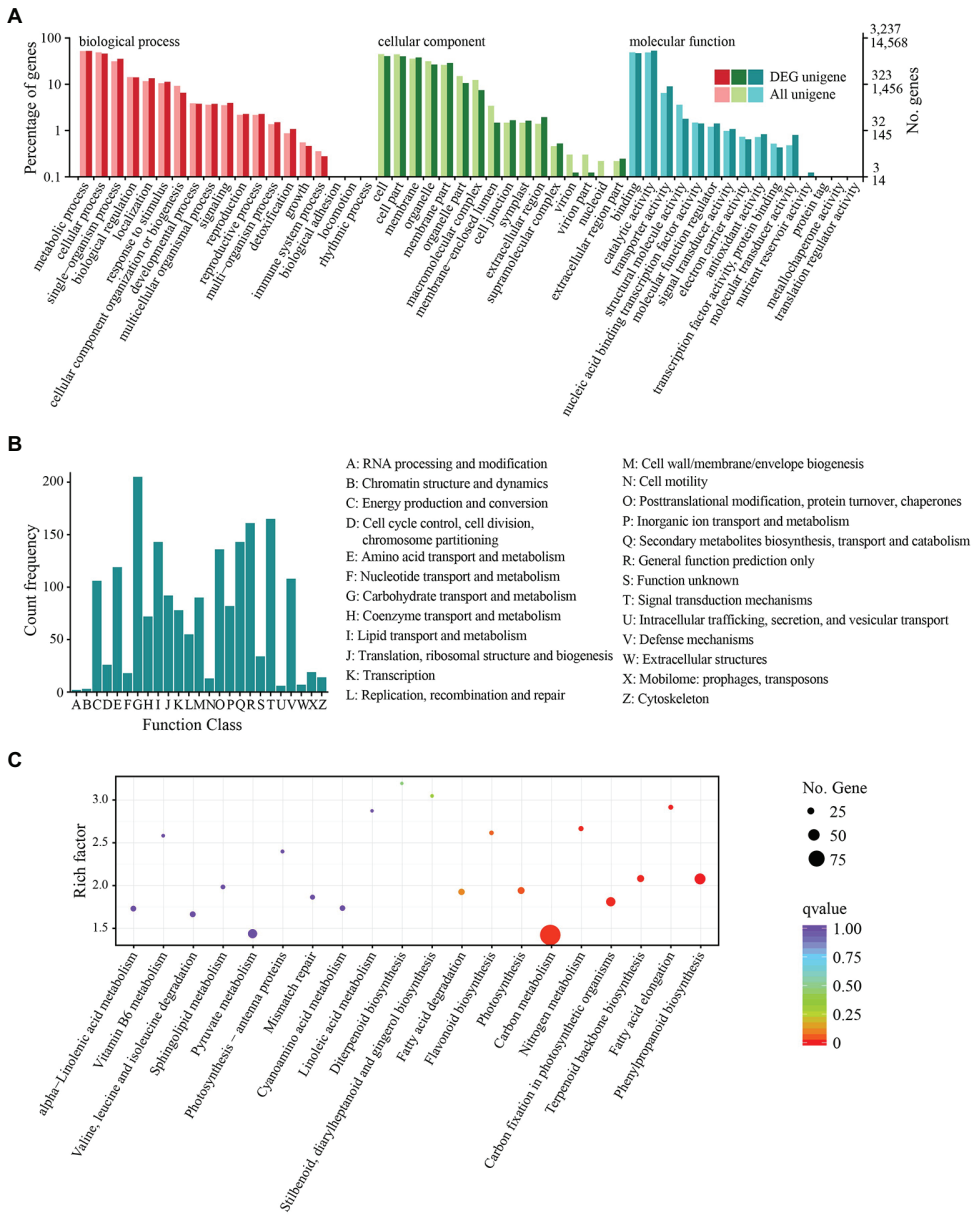
Functional and pathway enrichment of all DEGs. **(A)** GO enrichment with three primary classifications: biological process, cellular component, and molecular function. **(B)** COG functional classification. **(C)** KEGG pathway enrichment. Each dot represents a KEGG pathway.

**Figure 3:**
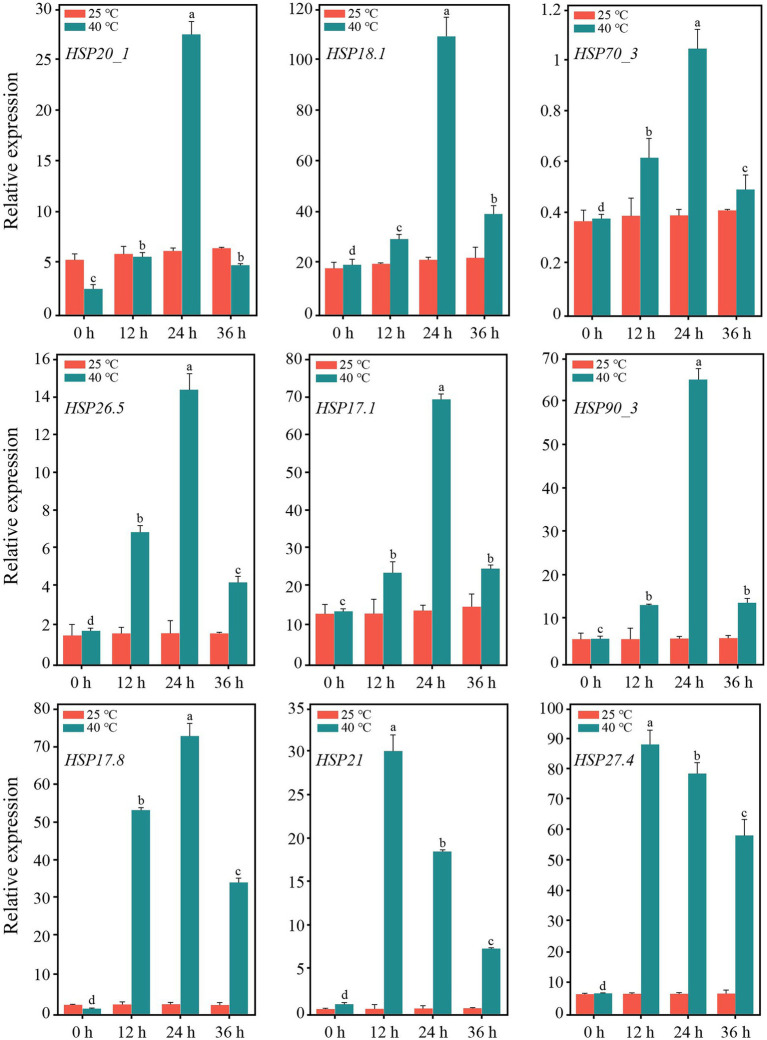
Expression of nine *PsHSP*s along with the time of high-temperature treatment. Different letters indicate that the expression level is significantly different at 40°C (*p* < 0.05) using a *t*-test. Three biological and technical replicates were set.

After 12 h of heat stress, 7,673 genes in TE were differentially expressed, with 4,220 upregulated and 3,453 downregulated genes compared to CK (Supplementary Figure S5). Among the three primary classifications of GO functional enrichment, 6,443 DEGs were enriched in the biological process, mainly concentrated in the metabolic process (1702; 26.42%), cellular process (1,489; 23.11%), and single-organism process (1,148; 17.82%). There were 6,491 DEGs enriched in the cellular component, mainly in the cell (1,316; 20.27%), cell part (1,302; 20.06%), and membrane (1,232; 18.98%). In addition, 3,812 DEGs were enriched in molecular function, of which the majority were distributed in binding (1,523; 39.95%), catalytic activity (401; 45.09%), and transport activity (291; 7.63%). Overall, the immune system process, nucleoid, and nutrient reservoir activity were the least enriched (Figure 2A). Among the functional classifications in COG, 1897 DEGs were categorized into 26 groups. The most abundant annotations were enriched in carbohydrate transport and metabolism (G, 205; 10.81%), signal transduction mechanisms (T, 165; 8.70%), and general function prediction (R, 161; 8.49%), while the least abundant annotations were enriched in chromatin structure and dynamics (B, 3; 0.15%) and RNA processing and modification (A, 2; 0.11%; Figure 2B). For KEGG pathway enrichment, a total of 949 DEGs were annotated in 123 pathways, in which carbon metabolism was the most highly enriched pathway containing 99 DEGs, followed by 47 DEGs enriched in phenylpropanoid biosynthesis and 41 DEGs enriched in glycolysis/gluconeogenesis (Figure 2C). Among the DEGs, the expression of 18 *PsHSP*s was significantly upregulated in TE compared to CK (Supplementary Figures S5, S6), of which nine were further confirmed using qRT-PCR to verify the changes in time-dependent expression at 25°C and 40°C. At 25°C, the expression of these *PsHSP* genes was stable. After stress treatment at 40°C, all nine *PsHSPs* could respond to heat stress, with expression patterns rising first and then falling. The expression of *HSP20_1*, *HSP18.1*, *HSP70_3*, *HSP26.5*, *HSP17.1*, *HSP90_3*, and *HSP17.8* reached their maximum value after 24 h, while that of *HSP21* and *HSP27.4* reached the maximum value after 12 h (Figure 3).

### *sHSP* sequence characterization

**Figure 4:**
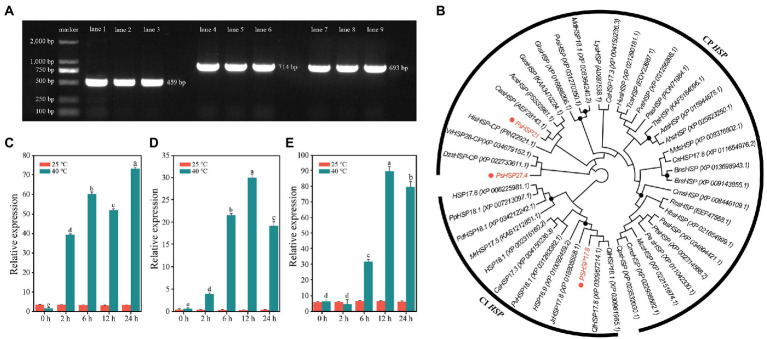
Sequence clones and analyses of three *PsHSP*s. **(A)** PCR products of the three *PsHSP*s. Lanes 1–3, 4–6, and 7–9 indicate amplified bands of *PsHSP17.8*, *PsHSP21*, and *PsHSP27.4*, respectively. **(B)** Neighbor-joining tree of three *PsHSP*s and *HSP*s of other species with accession numbers in parentheses. Black dots at nodes indicate a bootstrap value <75. **(C–E)** Quantitative expression of *PsHSP17.8*, *PsHSP21*, and *PsHSP27.4* after high-temperature treatment at different times. Different letters indicate that the expression level at 40°C is significantly different (*p* < 0.05) using a *t*-test. Three biological and technical replicates were set.

Three *sHSP* genes with the highest fold change in differential expression, i.e., *PsHSP17.8*, *PsHSP21*, and *PsHSP27.4*, were cloned to obtain the complete cDNA sequences, of which the open reading frames (ORFs) were 459, 714, and 693 bp, respectively (Figure 4A). Therefore, they encoded 152, 237, and 230 amino acids (aa), respectively. All three were hydrophilic proteins without transmembrane regions or signal peptides (Supplementary Table S1; Figures S5A,C,D).

Based on motif functional site analyses, *PsHSP17.8* contained one protein kinase C (PKC) phosphorylation site and four casein kinase 2 (CK2) phosphorylation sites. An HSP20 feature alpha crystal domain (ACD) was found at 18–135 aa. *PsHSP20* contained one PK phosphorylation site and three CK2 phosphorylation sites, and the ACD was 129–237 aa. *PsHSP21* was predicted to contain three N-linked glycosylation sites, one cyclin-dependent kinase (CDK) phosphorylation site, two CK2 phosphorylation sites, and six PKC phosphorylation sites, and an *sHSP* domain was detected at 116–230 aa. All three *PsHSP* genes contained the feature domain ACD (HSP20) of the *sHSP* family, which was the same as the predicted results in NCBI (Supplementary Figure S7B). According to BLAST, *PsHSP17.8* was a cytosolic (CI) *sHSP* homologous to sHSP-Class I in other species, while *PsHSP21* and *PsHSP27.4* were homologous to chloroplast (CP) *sHSP*s, and both were organelle *sHSP* genes. The prediction of protein structures showed that *PsHSP17.8*, *PsHSP21*, and *PsHSP27.4* contained 3, 4, and 4 α-helix structures, 10, 14, and 14 *β*-sheet structures, 28, 21, and 37 turn structures, and 24, 18, and 25 curvilinear loop structures, respectively (Supplementary Figure S8). Amino acid sequence alignment also revealed that all three *sHSP*s contained *HSP20* conserved domains with nine conserved *β*-sheets (*β2*–*β10*) at the variable N-terminal and the conserved C-terminal, which were consistent with the α crystallin conserved domains (Supplementary Figure S9). The phylogenetic tree resolved *PsHSP17.8* close to *QlHSP17.8* and *QlHSP18.1* (*Quercus fabri*) within the CI clade. Within the CP clade, *PsHSP21* was close to *CasHSP* (*Chenopodium album*), while *PsHSP27.4* was closest to *DzsHSP* (*Durio zibethinus*) in another clade that diverged from *PsHSP27.4* (Figure 4B). Compared with the control, *PsHSP17.8*, *PsHSP21*, and *PsHSP27.4* were all rapidly upregulated within 24 h to respond to heat stress. *PsHSP17.8* and *PsHSP21* were significantly upregulated at 2 h after high-temperature treatment, while *PsHSP27.4* was upregulated at 6 h (Figures 4C–E).

### Functional analyses of *sHSPs* in transgenic lines

**Figure 5:**
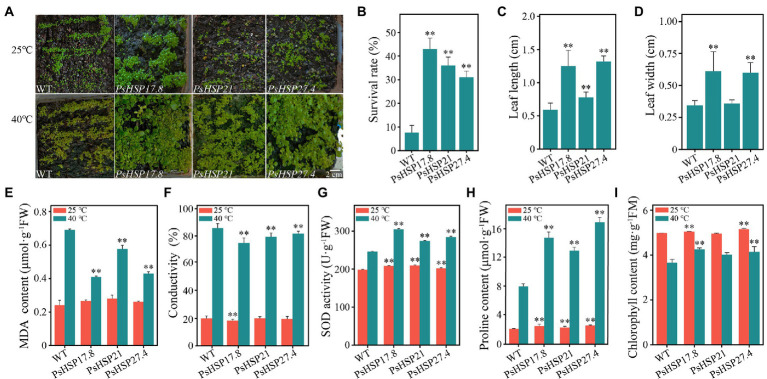
Growth situations and several indices of transgenic lines after 21-day recovery from acute heat stress at 40°C for 4 h. **(A)** Growth of transgenic lines and the WT. **(B)** Survival rate of TE and CK after recovery from heat stress. **(C)** Differences in leaf length between transgenic lines and the WT. **(D)** Differences in leaf width between transgenic lines and the WT. **(E–I)** MDA content, conductivity, SOD activity, proline content, and chlorophyll content of transgenic lines and the WT. ^**^ indicates significant differences compared to the WT at 25°C and 40°C (*p* < 0.05) using a *t*-test. Three biological and technical replicates were set.

The expression of *PsHSP17.8*, *PsHSP21*, and *PsHSP27.4* in different tissues of transgenic lines (MT) was detected using qRT-PCR, which elucidated that the three *sHSPs* were expressed more in leaves and less in flowers and siliques. In addition, the expression levels of *PsHSP21* and *PsHSP27.4* in the stem were also higher than those of *PsHSP17.8* (Supplementary Figure S10). The overexpressed transgenic lines and wild-type (WT) seedlings were stressed at 40°C for 4 h. After the 21-d recovery, the overall growth and development of the three types of transgenic lines were much better than that of the WT (Figure 5A). The survival rates of *PsHSP17.8*, *PsHSP21*, and *PsHSP27.4* reached 43, 31, and 36%, respectively, after a 21-day recovery from acute heat stress, despite a few plants showing leaf discoloration and leaf margin discoloration (Figure 5B). The leaf lengths of *PsHSP17.8* (1.25 ± 0.24 cm), *PsHSP21* (0.78 ± 0.08 cm), and *PsHSP27.4* (1.32 ± 0.09 cm) were 2.1, 1.31, and 2.2 times higher than that of the WT (0.59 ± 0.10 cm), respectively, and the leaf widths of *PsHSP17.8* (0.61 ± 0.15 cm) and *PsHSP27.4* (0.60 ± 0.08 cm) were also higher than those of *PsHSP21* (0.36 ± 0.03 cm) and the WT (0.34 ± 0.04 cm). There was no significant phenotypic difference between the leaves of the overexpressed *PsHSP17.8* and *PsHSP27.4* lines, but they were both longer than those of the *PsHSP21* line (Figures 5C,D). There were also significant changes in the physiological and biochemical indices between the transgenic lines and the WT.

The MDA content (0.24 ± 0.01–0.28 ± 0.01 μmol·g^−1^FW), conductivity (18.18 ± 1.00–19.78 ± 1.21), SOD activity (198.18 ± 4.70–209.77 ± 9.73 U·g^−1^FW), proline content (2.13 ± 0.04–2.57 ± 0.10 μmol·g^−1^FW), and chlorophyll content (4.95 ± 0.19–5.52 ± 0.02 mg·g^−1^FM) remained relatively stable in both the WT and the three transgenic lines (Figures 5E–I). After acute heat stress, the MDA content (0.41 ± 0.02, 0.58 ± 0.01, and 0.43 ± 0.02 μmol·g^−1^FW, respectively) and conductivity (73.81 ± 3.70, 78.25 ± 2.81, and 80.58 ± 1.78, respectively) of the *PsHSP17.8*, *PsHSP21*, and *PsHSP27.4* transgenic lines were significantly lower than those of the WT (0.69 ± 0.02 μmol·g^−1^FW and 86.23 ± 3.10), indicating that the WT experienced more intense membrane lipid peroxidation (Figures 5E,F). The SOD activity (304.67 ± 16.92, 274.11 ± 15.53, and 284.21 ± 9.12 U·g^−1^FW) in the transgenic lines was significantly enhanced compared to MT (246.01 ± 12.53 U·g^−1^FW; Figure 5G). The free proline content in the transgenic lines was significantly increased (14.76 ± 0.78, 12.96 ± 0.46, and 16.89 ± 0.69 μmol·g^−1^FW) compared to the WT (7.98 ± 0.38 μmol·g^−1^FW; Figure 5H). In addition, the chlorophyll content in both WT and transgenic lines was also affected after heat treatment and decreased to varying degrees. However, the WT showed the greatest decrease from 4.97 ± 0.13 to 3.66 ± 0.08 mg·g^−1^FM (Figure 5I), while the decrease in *PsHSP17.8*, *PsHSP21*, and *PsHSP27.4* transgenic lines was from 5.05 ± 0.26 to 4.25 ± 0.10, from 4.95 ± 0.19 to 4.01 ± 0.15, and from 5.52 ± 0.02 to 4.14 ± 0.12 mg·g^−1^FM, respectively.

## Discussion

### Multiple DEGs, especially *HSP*s, involved in responses to heat stress in tree peony

In this research, after heat stress, the annotation of DEGs in the GO functional group mainly concentrated on metabolic and cellular processes, which were similar to that of tea (*Camellia sinensis*) and passion fruit (*Passiflora edulis*; Izzo and Schneider, 2016). In many cases, stress causes the accumulation of secondary metabolites (Xu et al., 2016; Qi et al., 2021). KEGG enrichment revealed that some DEGs were involved in phenylpropanoid biosynthesis and terpenoid backbone biosynthesis pathways, and 59 DEGs were involved in endoplasmic reticulum protein processing, including members of *SEC*s, *UBC*s, and *HSP*s (Figure 2). It has been widely demonstrated that heat stress not only affects normal protein processing and folding in the endoplasmic reticulum but also leads to excessive accumulation of unfolded proteins (Wahid et al., 2007). In addition, 21 DEGs were annotated to CDPK-related pathways, such as the metabolic pathways of glycine, serine, and threonine. This suggests that CDPK family members could act as important elements in the transduction network in response to heat stress in tree peony. Relevant research in *Populus* (*P. euphratica*) showed that *PeCPK10* could act as an effective heat-tolerant gene to reduce damage and protect organic tissues during heat stress (Chen et al., 2013). These results indicated that the changes in carbon metabolism, biosynthesis, and endoplasmic reticulum protein processing pathways are actively involved in tree peony response to heat stress. In addition, under heat stress, photosynthesis and respiration will cause oxidative stress and ROS accumulation, which will destroy the balance and stability of the intracellular environment. Plants contain enzymatic reactions and non-enzymatic reactions to clear redundant ROS in time. Here, several DEGs were enriched in the glutathione metabolic pathway, indicating that glutathione, as a non-enzymatic reactive oxygen scavenging mechanism, played an important regulatory role in the heat stress of tree peony. The upregulated expression of *HSP*s is closely related to environmental stress (Chen et al., 2018). *HSP*s participate in the folding of cellular proteins and the protection of important functional activities in the body. The accelerated expression of *HSP*s in plants is a ubiquitous response to heat stress and is also a coping strategy for adapting to the environment and reducing or mitigating damage (Hendrick and Hartl, 1995; Sun et al., 2002; Huang and Xu, 2008). As major stress response proteins, *HSP*s can act synergistically with other regulatory processes, in addition to their direct role in stress tolerance, both to reduce cellular damage in the organism (Wang et al., 2004b). After the occurrence of heat stress, the denaturation of proteins accelerates, together with the random aggregation and the degradation of physiological activities. The several differentially expressed *PsHSP* genes found here were all upregulated under heat stress in tree peony (Figures 3, 4D). The spatiotemporal expression revealed by the qRT-PCR analyses also showed that, under acute high temperatures, the expression of *PsHSP*s increased with increasing stress time. The high expression of *PsHSP*s in a short time indicated rapid responses to heat stress involving the regulation of *HSP*s in tree peony, which was also shown in the transgenic lines (Figure 4). Plenty of chaperones, including *HSP*s, can participate in the response to diverse abiotic stress, are involved in regulating the signaling pathways, and can help protein folding under stress, enabling the plant to avoid harm since it is fixed on the ground and cannot actively escape (Jacob et al., 2017), and contribute to the assembly, translocation, and degradation of proteins to balance cellular homeostasis (Liu et al., 2018). In the *HSP* family of higher land plants, in addition to ATP-dependent high molecular weight *HSP*s larger than 60 kDa, the members of ATP-independent low molecular weight *HSP*s are still complex and diverse, which will act specifically in massive processes, including temperature responses, regulation of photosynthetic efficiency, mitigation of ionic damage, and protection of plant organelles (Wahid et al., 2007).

### *sHSP*s improved heat resistance, affecting changes in physiological and biochemical responses

Heat stress interferes with normal physiological processes. For instance, the aberrant accumulation of ROS, including MDA, O_2_^−^, and -OH disturbs the balanced biosystem and causes oxidative damage to lipids, nucleic acids, proteins, and cell membranes (Mittler et al., 2012; Suzuki et al., 2012). Normally, trace damage caused by oxidation can be effectively alleviated through redox reactions and metabolic processes. When under stress, organisms mobilize various antioxidant enzymes to reduce damage once they sense this stimulus. Additionally, the responses of organelles (chloroplasts, mitochondria, and others) to oxidative stress triggered by ROS generate secondary stress. This secondary stress is characterized by the emergence of massive ROS from both endogenous and exogenous accumulation (Gill and Tuteja, 2010; Jalmi and Sinha, 2015). Although antioxidant enzymes such as SOD can scavenge some O_2_^−^, their ability is limited. The *HsfA4a* protein is a signal sensor of exogenous H_2_O_2_ and can induce the expression of THs, along with the activation of the mitogen-activated protein kinase (MAPK) signaling pathway at high temperatures. Downstream TFs, such as *ZATs*, *WRKYs*, and others, can stimulate the expression of antioxidant enzymes to reduce ROS signaling, and multiprotein bridging factor 1 (*MBF1*) acts as a transcriptional co-activator (Miller and Mittler, 2006). In addition, the accumulation of osmotic regulators under heat stress can strengthen the stability of protein and membrane structures, such as carbohydrates, proline, and soluble proteins that accumulate under stress (Qu et al., 2013).

The functions of *sHSP*s in stress resistance primarily involve extreme temperature tolerance and high antioxidant capacity. Here, three *sHSP*s, i.e., *PsHSP17.8*, *PsHSP21*, and *PsHSP27.4*, could be induced by high temperature. Although the expression patterns of the three *sHSP*s were not completely consistent, they could be stimulated by high-temperature signals within a very short period of 2–12 h (Figure 4), indicating that the expression of *sHSP*s in tree peony could be rapidly increased to exert the functional activity of stress proteins to stabilize normal proteins, prevent protein refolding, and degrade abnormal proteins. The MDA content and conductivity of the *sHSP* transgenic lines were lower than those of the WT, while the SOD activity, free proline content, and chlorophyll content were significantly higher after heat stress (Figures 5E–I). Conductivity can indicate the degree of membrane damage, and MDA can indicate the degree of lipid peroxidation. The MDA content of the three transgenic lines also decreased to varying degrees. The accumulation of ROS can be caused by the increased lipid peroxidation of plant cell membranes. The SOD activities of the three transgenic lines were higher than those in the normal-temperature treatment. As one of the protein components, the accumulation of free proline indicated that these substances were involved in regulating the intracellular osmotic potential and stabilizing the cell structure. The transformation of *PsHSP17.8*, *PsHSP21*, and *PsHSP27.4* could improve the tolerance of *A. thaliana* to heat stress. The leaf length and width of the transgenic lines were larger than those of the WT after high-temperature treatment (Figures 5A,C,D), indicating that the overexpressed lines of *PsHSP17.8*, *PsHSP21*, and *PsHSP27.4* could be more tolerant than the WT after high-temperature treatment. *HSP17.8* can act as a cofactor for the Ankyrin-repeat containing protein 2A (*AKR2A*) in unstressed cells and plays an important role in targeting the chloroplast (Kim et al., 2011; Hu et al., 2020). The loss of *HSP21* resulted in white leaves in *A. thaliana*, which could recover to green by overexpression of *HSP21* (Zhong et al., 2013). *HSP27* can improve heat tolerance (Wang et al., 2004a). However, the mechanisms by which *PsHSP17.8*, *PsHSP21*, and *PsHSP27.4* respond to high temperatures and regulate the heat tolerance of tree peony remain unknown. To clarify the expression patterns through subcellular localization, investigating their initiations and key TFs is necessary to study the regulatory mechanism of *sHSP*s in tree peony for a comprehensive understanding of the molecular regulatory mechanism of heat tolerance.

## Conclusion

**Figure 6:**
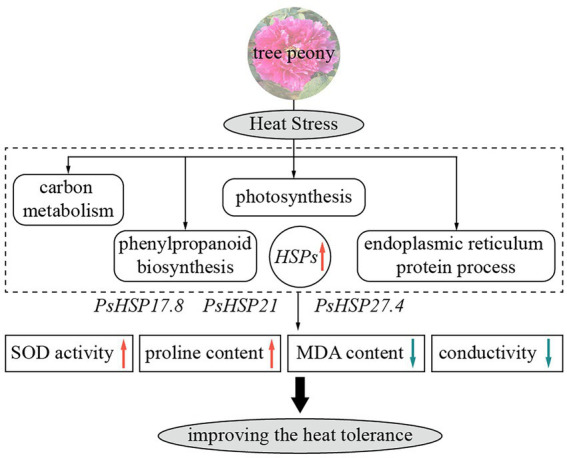
Schematic diagram of heat responses and functions of three *sHSP*s in tree peony showing major relevant changes. Red and blue arrows indicate upregulation and downregulation, respectively.

Against the background of increasing global warming, it is essential to explore the effects of high temperatures on cool-adaptative ornamental plants and, in turn, the physiological, biochemical, and molecular responsive mechanisms of plants to heat stress. In this research, based on RNA-seq, we compared the transcriptomic changes in peony leaves before and after heat stress. Several processes and pathways were related to the heat resistance of tree peony, including the metabolic process, cellular process, single-organism process, phenylpropane biosynthesis pathway, endoplasmic reticulum protein synthesis pathway, photosynthesis pathway, and carbon metabolism pathway. The functions of three important *sHSP*s, i.e., *PsHSP17.8*, *PsHSP21*, and *PsHSP27.4*, were related to heat stress in tree peony, as their overexpression could preliminarily improve the thermotolerance of transgenic lines by changing relevant metabolic content (Figure 6). Our results will provide molecular and genetic resources for further research involving the heat tolerance and innovation of cultivated tree peony.

## Data availability statement

The data presented in the study are deposited in the GenBank repository (https://www.ncbi.nlm.nih.gov/ accession numbers OM489532, OM489533, and OM489534; Bioproject repository, accession number PRJNA802352).

## Author contributions

JM, JW, and QW performed the experiments. LS, YZ, GZ, and QM performed transcriptomic analysis. JM, JW, QW, SH, and CG wrote and reviewed the manuscript. CG provided the funds. All authors contributed to the article and approved the submitted version.

## Funding

This research was supported by grants from Zhejiang Provincial Natural Science Foundation of China (grant no. LY21C160001).

## Conflict of interest

The authors declare that the research was conducted in the absence of any commercial or financial relationships that could be construed as a potential conflict of interest.

## Publisher’s note

All claims expressed in this article are solely those of the authors and do not necessarily represent those of their affiliated organizations, or those of the publisher, the editors and the reviewers. Any product that may be evaluated in this article, or claim that may be made by its manufacturer, is not guaranteed or endorsed by the publisher.

## Footnotes

^1^ https://www.lynnon.com/dnaman.html

^2^ https://genome.cbs.dtu.dk/services/TMHMM-2.0
