# Supplementary material for: Physiological and transcriptional responses to heat stress and functional analyses of PsHSPs in tree peony (Paeonia suffruticosa)
Source: Front Plant Sci. 2022 Aug 11;13:926900. doi: 10.3389/fpls.2022.926900 (PMC9403832; doi:10.3389/fpls.2022.926900)
Supplement: Supplementary file 1 [file Data_Sheet_1.docx]

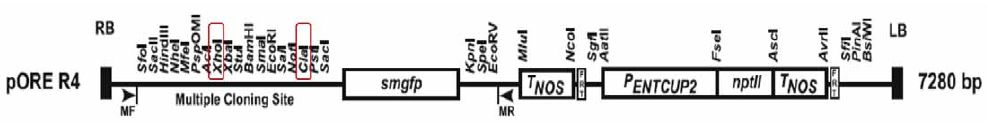


Fig. S1. Plant overexpression vector *pORE_R4* map. The red circle indicates the two selected restriction sites.


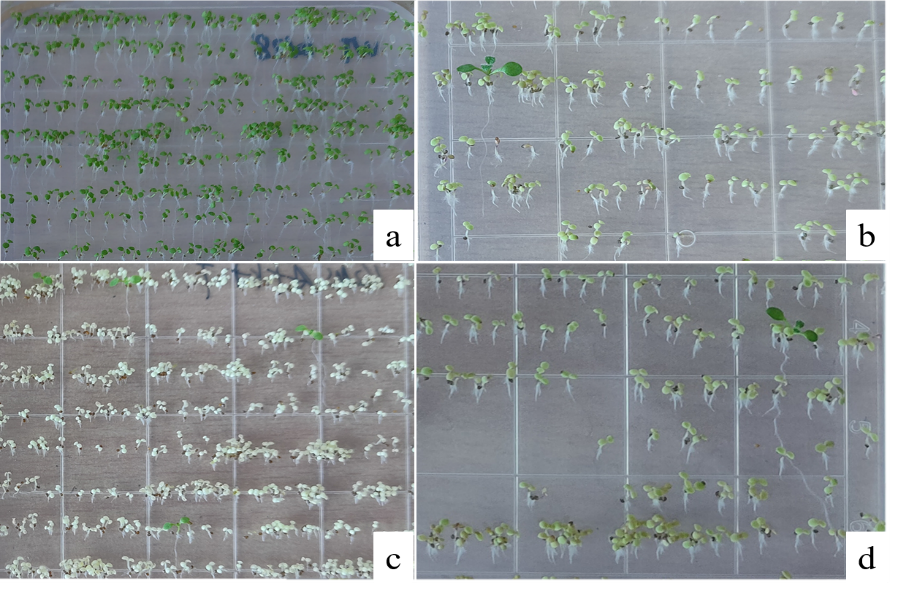


Fig. S2. Positive screening of transgenic lines. (a) WT strain, (b) *PsHSP17.8* strain, (c) *PsHSP21 strain*, (d) *PsHSP27.4* strain.


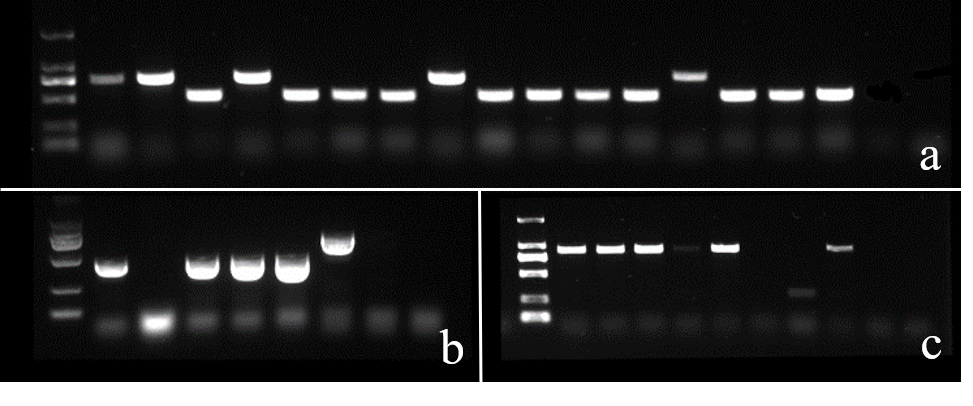


Fig. S3. DNA genetic identification of transgenic lines. (a-b) *PsHSP17.8* and *PsHSP21* strains, and © is PsHSP27.4 strain.


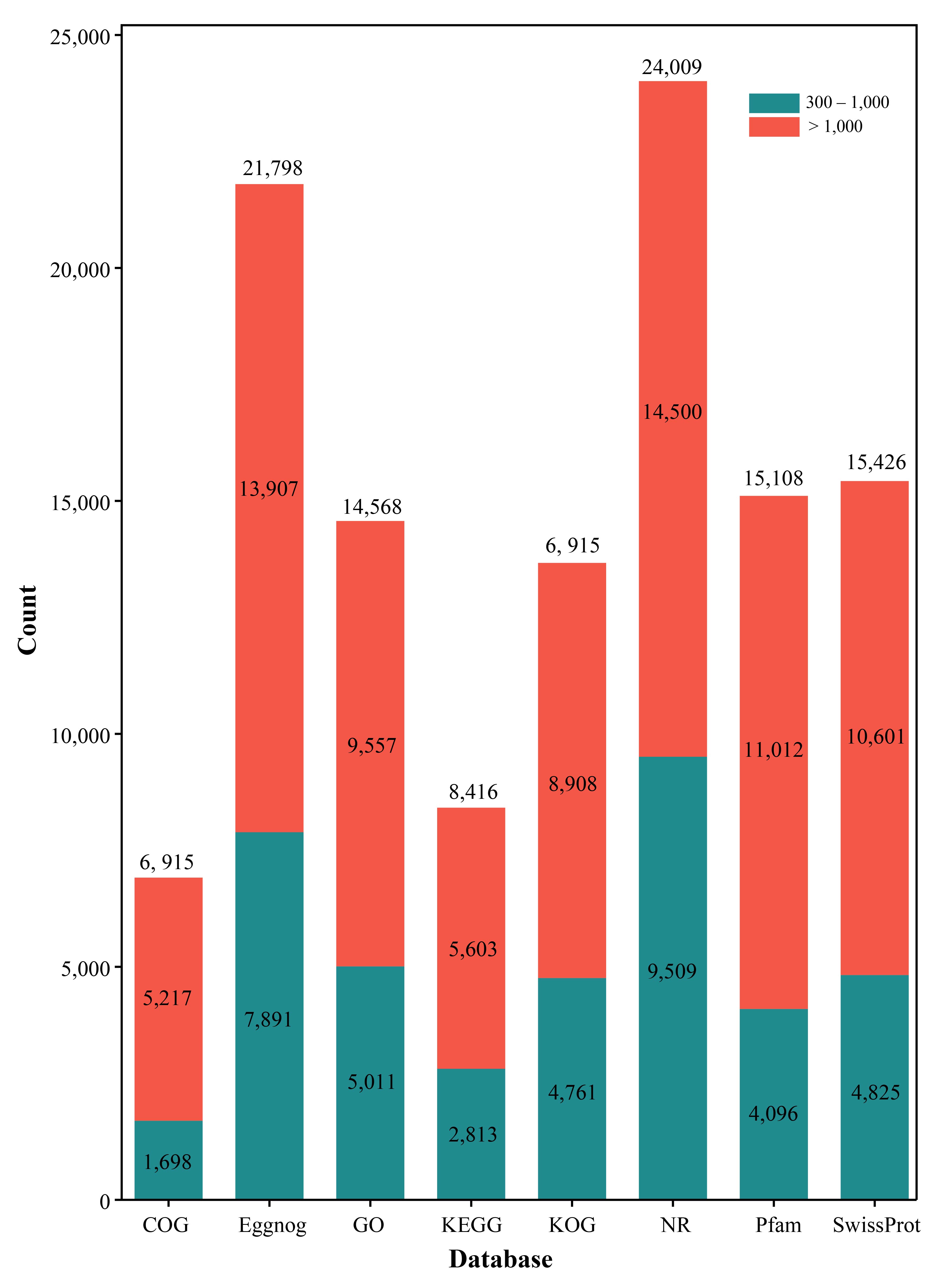


Fig. S4. The number of unigene annotations from eight databases. The number of the total, 300 –1,000 bp, and > 1,000 bp annotated unigenes were labeled from top to bottom of bars.


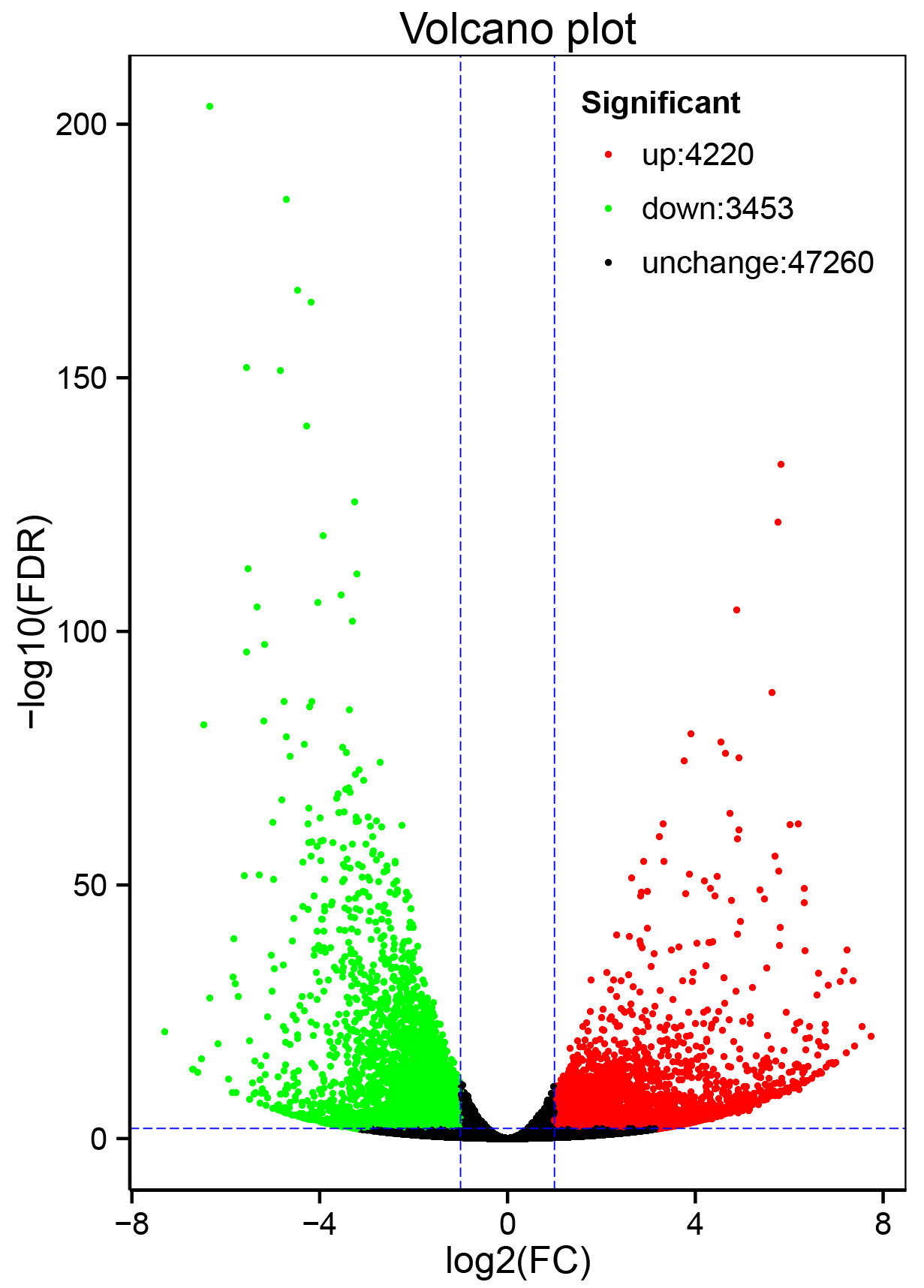


Fig. S5. The volcano plot of TE vs. Ck. Each point represented a gene. The red and green dots indicated up and down regulated genes, respectively, while the black dots indicated no significant difference in expression of the gene.


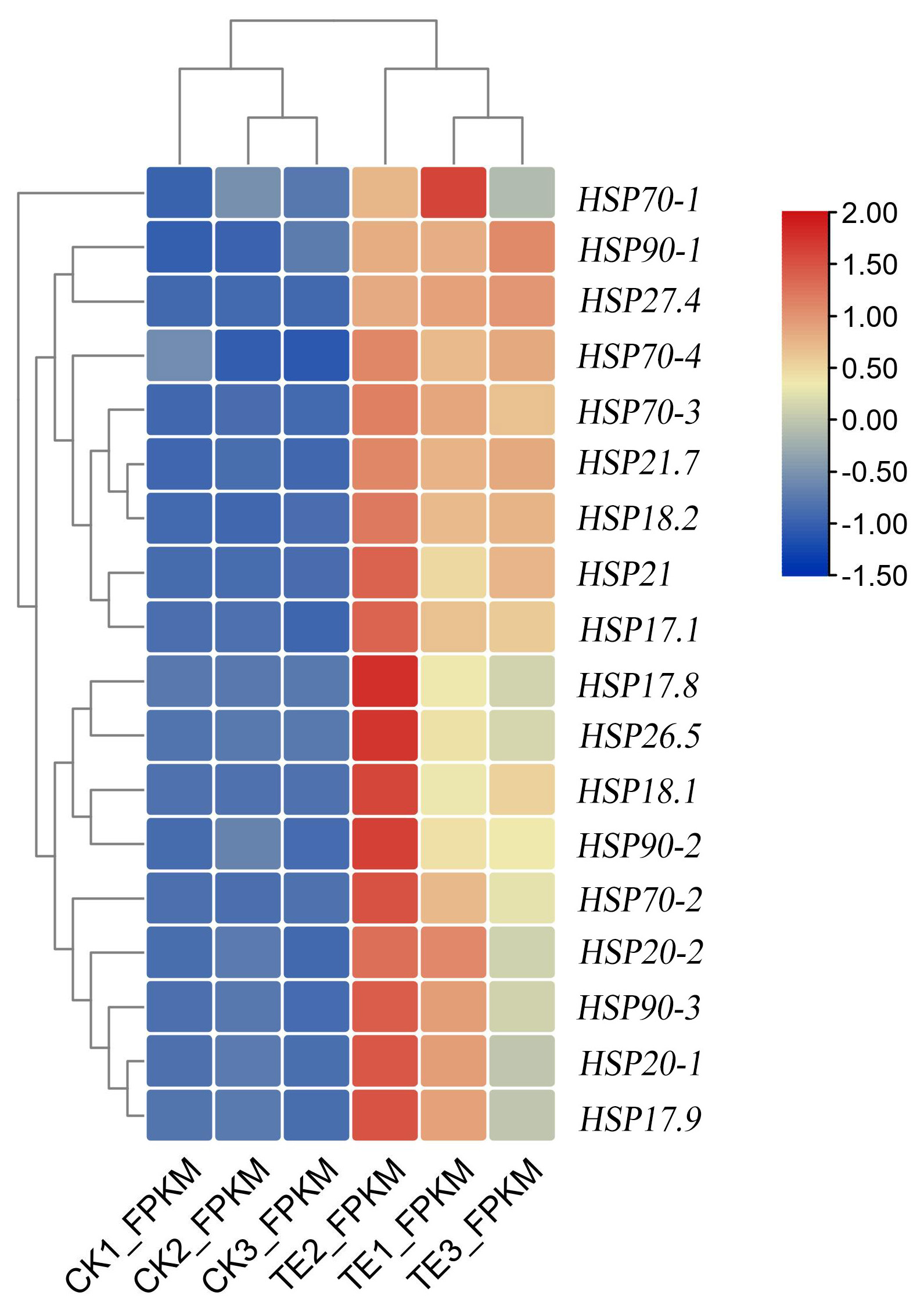


Fig. S6. The heatmap of 18 *PsHSP* expressions in the transcriptome data. CK were control groups at 25℃ and TE were treatment groups at 40℃.


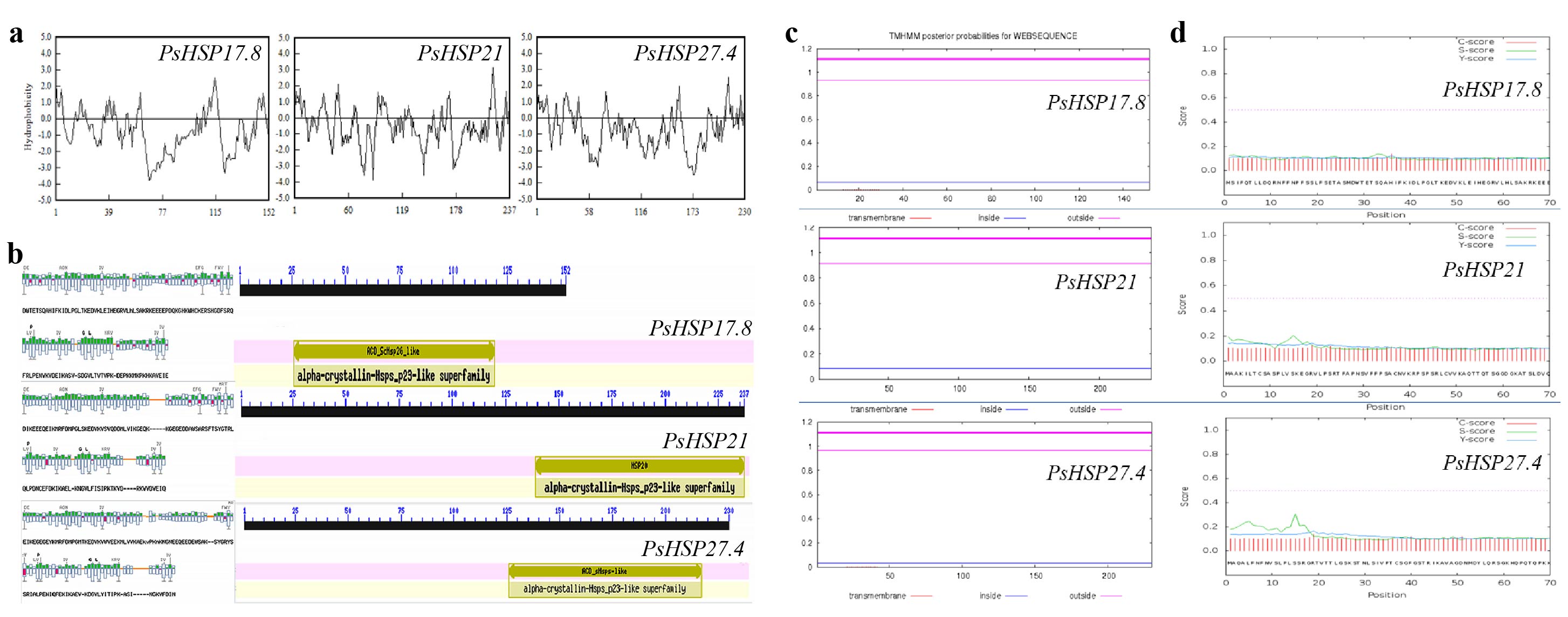


Fig. S7. Physicochemical analyses of three *PsHSP*s. (a) Hydrophobicity site map. (b) The specific ACD domain of HSP. (c) Transmembrane region analyses. (d) Signal peptide analyses.


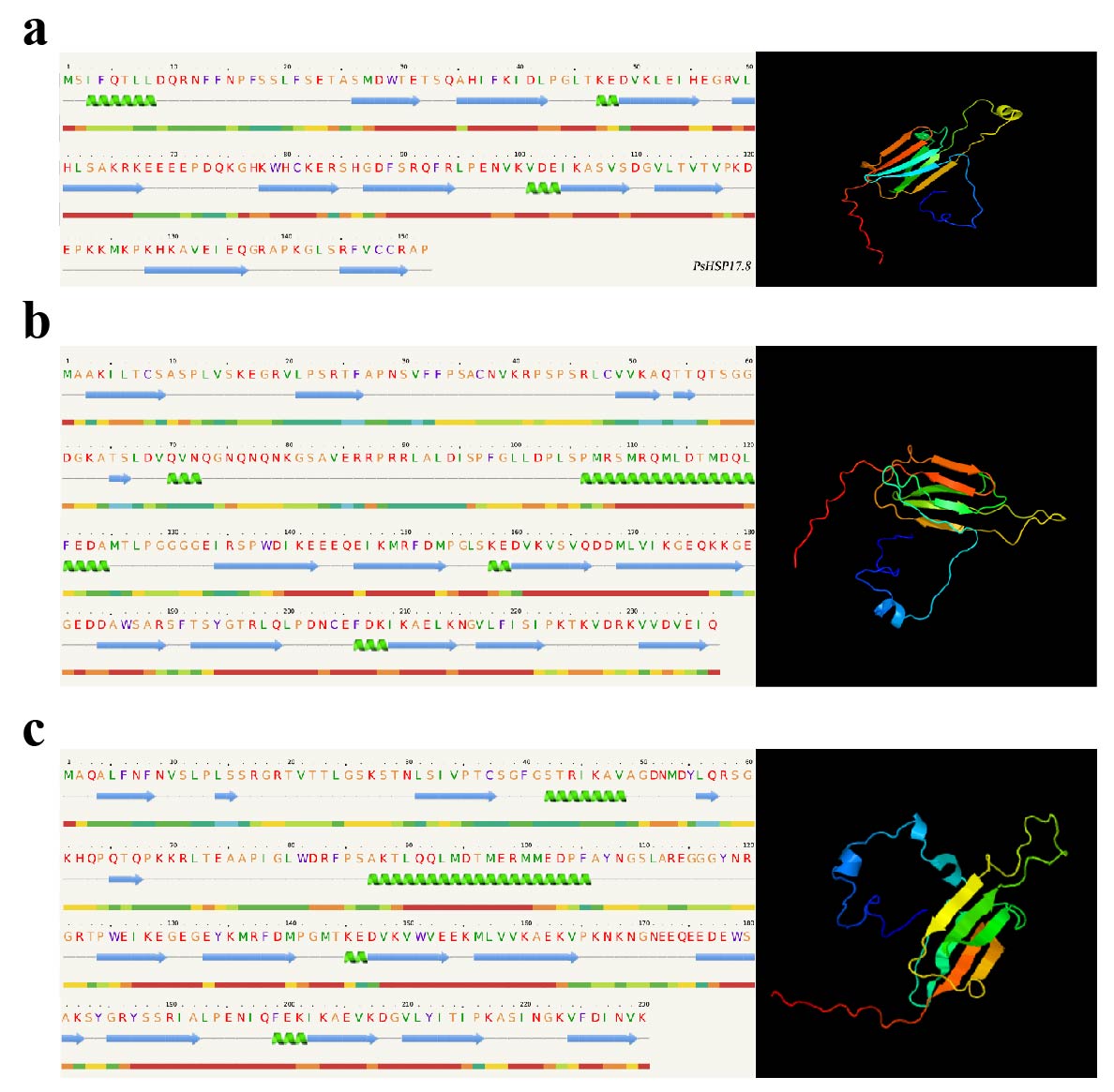


Fig. S8. Analyses of the secondary and tertiary structure of (a) *PsHSP17.8,* (b) *PsHSP21*, and (c) *PsHSP27.4*.


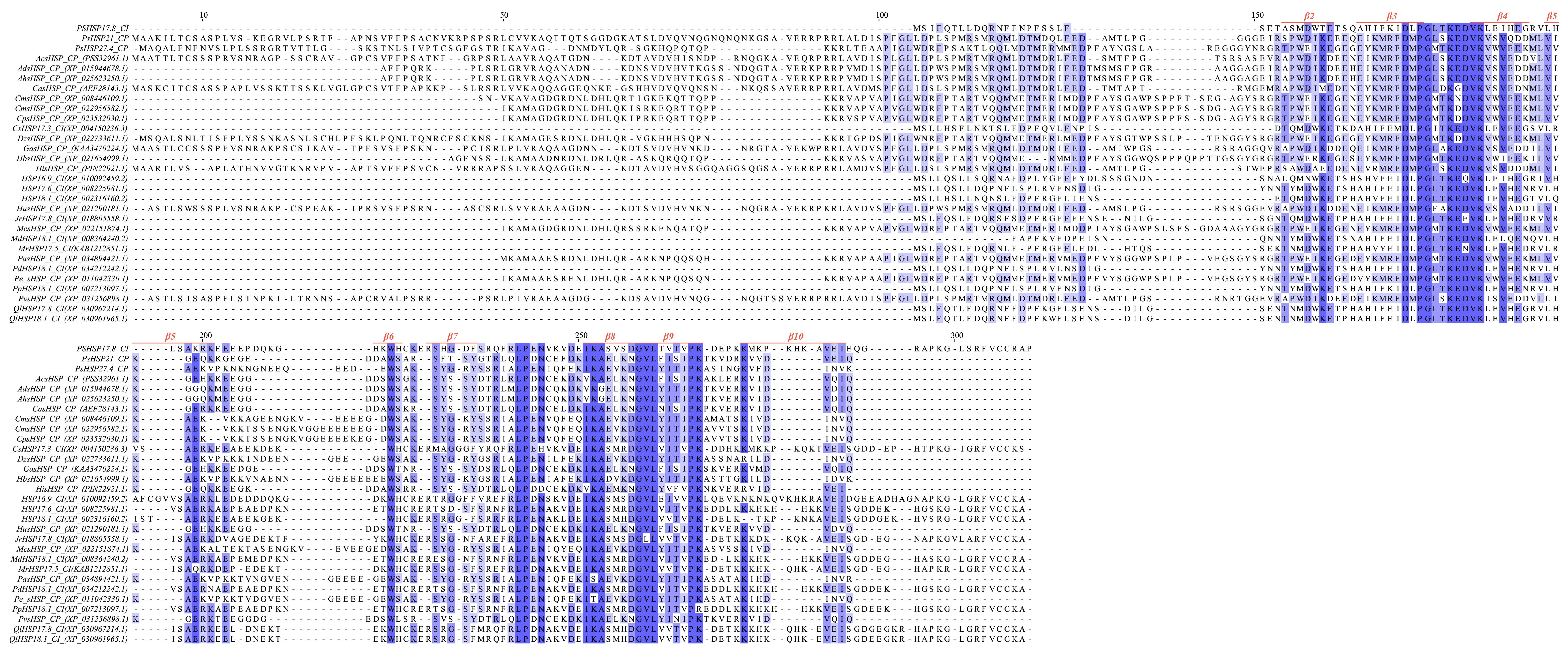


Fig. S9. The alignment of three *PsHSP*s and *HSP*s of other species. Nine conserved *β*-sheets (*β2* – *β10*) were showed by the red lines.


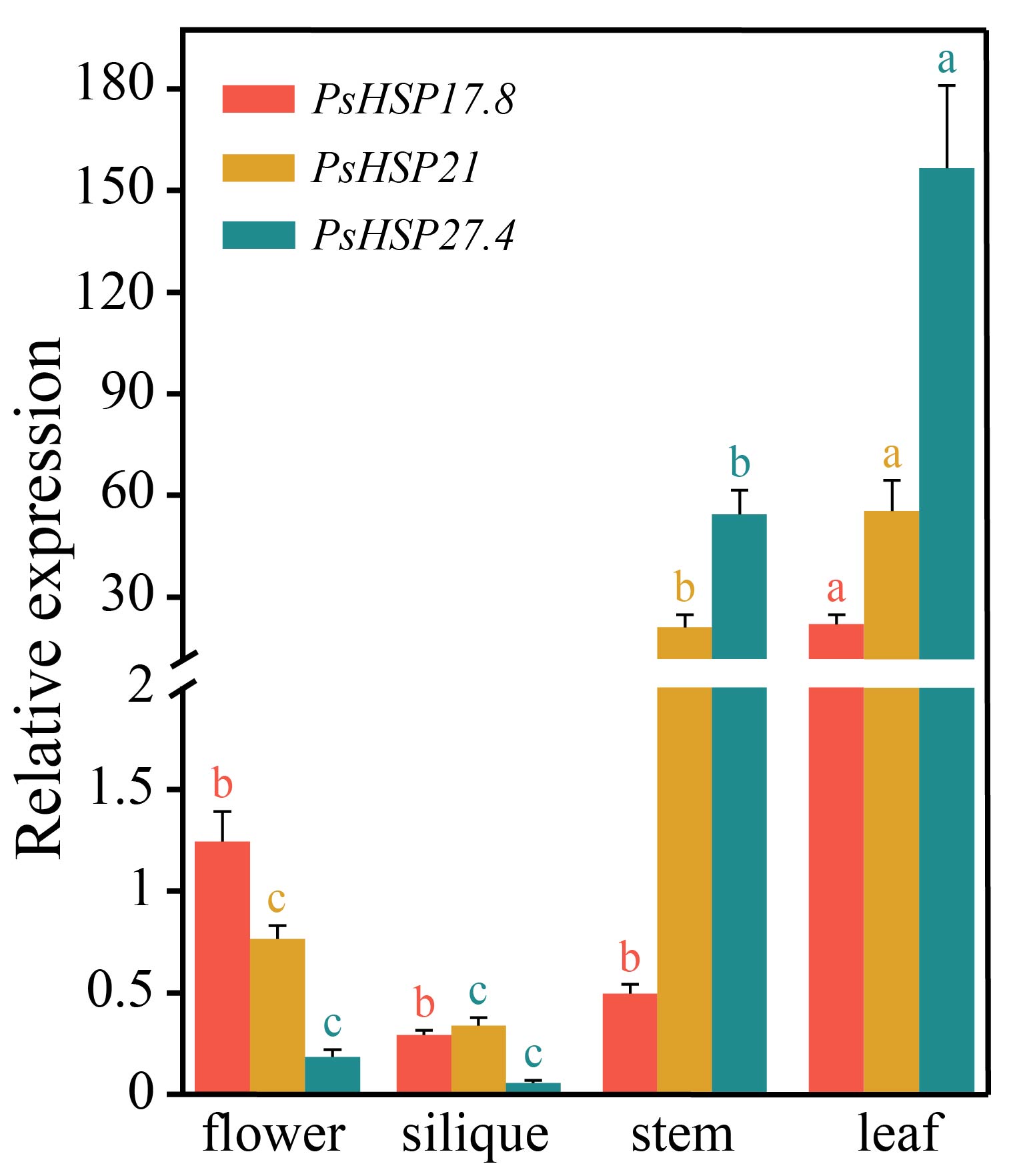


Fig. S10. Quantitative expression in different tissues of transgenic lines. Different letters indicated significance among the same tissue (*p* < 0.05).

Table S1. Characteristics of *PsHSP17.8*, *PsHSP21*, and *PsHSP27.4*.

|  | *PsHSP17.8* | *PsHSP21* | *PsHSP27.4* |
| --- | --- | --- | --- |
| ORF length (bp) | 459 | 714 | 693 |
| No. aa | 152 | 237 | 230 |
| Molecular weight (kDa) | 83.53 | 88.47 | 91.87 |
| Formula | C_3016_H_5035_N_999_O_1258_S_253_ | C_3207_H_5369_N_1047_O_1334_S_266_ | C_3389_H_5660_N_1120_O_1412_S_224_ |
| Theoretical isoelectric point | 5.04 | 4.96 | 5.07 |
| No. atom | 10,561 | 11,223 | 11,805 |
| Instability index | 53.35 | 51.26 | 37.64 |
| Fat soluble | 24.92 | 26.27 | 30.62 |
| Grand average of hydropathicity | -0.766 | -0.508 | -0.682 |
